# Feasibility of Using Resting Heart Rate and Step Counts From Patient-Held Sensors During Clinical Assessment of Medical Emergencies (FUSE): Protocol for Prospective Observational Study in European Hospitals

**DOI:** 10.2196/55975

**Published:** 2025-04-28

**Authors:** Jack Barrington, Christian Subbe, John Kellett, Erika Frischknecht Christensen, Mikkel Brabrand, Prabath Nanayakkara, Jelmer Alsma

**Affiliations:** 1 Department of Acute Medicine Ysbyty Gwynedd Bangor United Kingdom; 2 North Wales Medical School Bangor University Bangor United Kingdom; 3 School of Health and Social Care University of Bolton Bolton United Kingdom; 4 Centre for Prehospital and Emergency Research Aalborg University Hospital Aalborg Denmark; 5 Research Unit for Emergency Medicine Odense University Hospital Odense Denmark; 6 Amsterdam University Medical Centre Vree University Medical Centre Amsterdam The Netherlands; 7 Department of Internal Medicine Erasmus University Medical Center Rotterdam The Netherlands

**Keywords:** mHealth, emergencies, heart rate, step count, hospital admission, intensive care, medical emergency, smart watch, feasibility, clinical assessment, wearables, vital signs, distress, wearable sensors, mobility, device, mobility data

## Abstract

**Background:**

Abnormalities of vital signs are quantified by comparison with normal ranges, which are those observed in resting healthy populations. It might be more appropriate to compare the vital sign values of an individual in distress with their own usual values recorded when they were stable and well. Sensors from smartwatches or smartphones might make this possible at scale, but the proportion of patients using them is not known.

**Objective:**

This study aimed to assess the feasibility of using heart rate and mobility data from patients’ own wearable sensors as part of clinical assessments at the time of presentation to hospitals with medical emergencies, and to quantify the difference between heart rate and the change in daily steps taken by the patient on admission to acute care compared with the previously recorded values at home.

**Methods:**

This is an international, multicenter observational study using the flashmob research design. The study will recruit patients aged 18 years and older who present to emergency departments, acute medical departments, or ambulatory emergency care with an acute medical complaint. Main end points of the study include the proportion of patients assessed for an acute complaint who use wearable devices to record vital signs. The study will describe the population that uses devices that collect vital signs in terms of sex, age group, digital literacy, and the severity of illness on presentation (as measured by a standard set of vital signs and frailty). Trends in heart rate and step counts measured in the month before presentation to acute care services will be reported according to discharge or admission status. Data will be collected during a pilot phase and during a single week in centers across Europe.

**Results:**

The study has been registered and passed the required approvals in the Netherlands Medical Ethics Committee (MEC-2022-0795) and the United Kingdom Integrated Research Application System (IRAS 321129). Based on the results of a pilot study performed at a single site in the United Kingdom, a flashmob study has been concluded in hospitals throughout Europe in May 2024 and reported in 2025.

**Conclusions:**

With the increasing availability of consumer held devices able to record medically relevant information this study will provide information about the availability of these data for clinical use in a number of European settings.

**International Registered Report Identifier (IRRID):**

DERR1-10.2196/55975

## Introduction

### Background

The decision about the need of an individual to require hospital admission relies on the assessment of patients’ symptoms, signs, past medical history, medication use, diagnosis, social support at home [1-3], and a judgment about the severity of illness based on estimated risk of deterioration in the subsequent hours and days [4].

Abnormalities of vital signs are quantified by comparison with normal measurements on healthy individuals recorded during periods of physiological stability. Within these individuals, the normal measurements vary and are influenced by age, sex, body composition, medication use, and physical condition [5-7]. There is a relation between the size of change from a physiologically normal range and the number of vital signs affected by abnormality and the frequency of adverse events [8]. Abnormality might be scored with generic tools that can be applied to the majority of patients, such as the National Early Warning Score [9], or tools that are disease-specific, such as the CURB65 [10], Blatchford [11], and DECAF (Dyspnea, Eosinopenia Consolidation, Acidemia, and Atrial Fibrillation) [12]. However, these scores can under- or overestimate risk in individual patients [13].

Knowing the values of an individual patient’s vital signs while well might help clinicians to better understand and interpret their changes or trends [14], their relative degree of deviation from the patient’s own normal and get a better estimate of their personal severity of illness. Similarly, mobility has been suggested to be a vital sign [15] and could be easily quantified by step counts. Normal mobility has been shown to be a protective factor [15]. Therefore, the assessment and comparison of step count during illness might be of value.

The general public are increasingly using devices such as smartwatches and mobile telephones that can potentially measure and record vital signs such as heart rate, heart rhythm, and oxygen saturation. Currently, approximately 90% of the population in the United States [16] and 94% in the United Kingdom [17] are smartphone users. Vital signs are also increasingly being recorded at home using conventional sphygmomanometers and pulse oximeters, and these values, if recorded, might also be available.

The aim of this study is to assess the feasibility of using a patient as their own reference for vital signs by assessing the difference between vital signs on admission to acute care with their own vital sign values recorded by his or her wearable device when they are at home and well (Textbox 1).

### Objectives

The primary and secondary study objectives are described in Textbox 1.

**Primary and secondary objectives.**

**Primary objectives**

- To assess the feasibility of using data from patients’ own wearable sensors for assessments in acute care. Feasibility will be assessed by eligibility, recruitment, retention rates, and patients’ acceptability.

**Secondary objectives**

- To explore the proportion of patients assessed for an acute complaint who are wearing or carrying a device that collects data on vital signs (eg, smartwatch or other wearables).
- To describe the population who wear or carry devices that collect data on vital signs.
- To quantify the change in heart rate in patients admitted to acute care between measurements taken on presentation and previously recorded vital signs by a consumer-grade wearable while patients were stable and well.
- To quantify the change in step count in patients admitted to acute care between measurements taken shortly before presentation and previously recorded step counts by a consumer-grade wearable while patients were stable and well.
- To explore the association between trends in in vital signs with the disposition of patients (admission to a general ward, critical care area, or discharge home within 24 hours from assessment).

## Methods

### Study Design

A pilot study was performed at a single site in the United Kingdom to develop and test procedures for data collection and reporting during the previous 6 months. Based on the learning from this pilot a prospective, international, multicenter, observational study using the flashmob research design will be conducted [18]. Participating units will collect data from patients presenting with acute medical emergencies to emergency departments, acute medical units, and ambulatory emergency care. There will be no randomization, blinding, and treatment allocation.

### Population

Patients who present at the emergency department, acute medical department, and ambulatory emergency care unit in secondary and tertiary hospitals in a sample of hospitals in the Netherlands, United Kingdom, Denmark, and Switzerland with an acute medical complaint. Acute medical complaints are nonsurgical admissions commonly treated by internists such as acute physicians, cardiologists, pulmonologists, gastroenterologists, endocrinologists, geriatricians, and similar (Textbox 2).

**Inclusion and exclusion criteria.**

**Inclusion criteria**

Participants in this study must be able to give informed consent, have an acute presenting complaint, be aged 18 years or older, and use any of the following: smartwatch, activity tracker, or other wearable monitoring devices, or a smartphone that collects step counts or data from other wearable devices.

**Exclusion criteria**

Patients who are unable to give informed consent or endure an imminently life-threatening illness.

**Main study end point**

To quantify the proportion of patients assessed for an acute complaint who are wearing a device that collects data on vital signs (ie, heart rate and step count).

**Secondary study end points**

Secondary end points will focus on four areas of interest:

- The difference between the heart rate at rest from the wearable device 24 hours, 1 week, and 1 month before assessment by emergency practitioners compared with the first set of vital sign parameters measured on admission to acute care.
- The change in daily steps taken in the month before admission to acute care.
- To describe the population that is wearing devices that collect data on vital signs in terms of gender, age group, severity of illness as measured by a standard set of vital signs, level of education, and digital literacy and frailty.
- To explore the association of changes in heart rate from the community to assessment in acute care services with clinical end points such as admission to a general ward or admission to a critical care area or discharge home within 24 hours from the time of clinical assessment.

### Study Procedures

All patients aged 18 years and older who present to acute care will be asked if they wear a smartwatch, activity tracker, or wearable that measures heart rate.

Patients will be asked to give informed consent in line with the guidance from the participating countries. This is likely to include written informed consent in the Netherlands, Denmark, and the United Kingdom. Informed consent will be explicit about accessing data from patient-held wearables (Multimedia Appendix 1). Once consent has been obtained, initial data will be entered into the case report form, and the remaining items of the case report form will be completed on the day after the initial presentation or later (Textbox 3). Study documentation will include a guide for investigators to interrogate commonly used wearable devices (Multimedia Appendix 2). Parameters recorded will vary according to the different devices used but should at least include heart rate and step count.

**Data items were collected as part of the "Feasibility of Using resting heart rate and step counts from patient-held Sensors during clinical assessment of medical Emergencies" (FUSE) study.**

- Site ID is the unique number for each site.
- Participant ID is a consecutive number (no identifiable information is collected).
- Location is the location where the patient is included in the study (ie, emergency department, medical admissions unit, ambulatory emergency care, or other).
- Sex as reported by the patient (ie, men, women nonbinary, or unknown).
- Date of birth, which will be used to compute an age category.
- Date and time of first presentation, defined as the time that a patient presents at the location.
- Highest level of education is the patient finished highest level of education or the level of education the patient is currently following (ie, primary school, secondary school, and higher education).
- Digital literacy is assessed using 2 questions asking for confidence of using the internet and confidence of using mobile apps, which patients can answer using a 5-point Likert scale.
- Device is the device used to measure heart rate, and the brand and type will be recorded.
- Phone is the phone that patients use, and this will be used to retrieve the step count and heart rate data. The brand and type will be recorded.
- Heart rate on the device is the heart rate that is stored and measured by the device, and the resting heart rate (as presented by the device), the minimum, maximum, and mean or median (depending on the device) will be retrieved on three-time points, namely on presentation, within 24 hours before presentation and within 1 week (plus or minus 1 day) before presentation.
- Walking distance is the number of steps of exact distance (in kilometers or miles), either measured by the device of the phone. The daily walking distance of the 7 days before the presentation will be recorded.
- Frailty as quantified through the Clinical Frailty Scale [19].
- Mobility will be assessed as either normal or impaired. Mobility is considered impaired if the patient is unable to get onto the bed unaided, either from the ambulance gurney or whichever way they arrive.
- Date and time of the first set of vital signs is defined as the time that the first set of vital signs are measured.
- Heart rate on admission is the first heart rate measured during clinical examination.
- National Early Warning Score [9] as a standardized assessment of the severity of illness, based on the first full measurements of respiratory rate (per minute), oxygen saturation (in %), temperature (in °C), heart rate (per minute), systolic blood pressure (in mm Hg), AVPU (alert, verbal, pain, and unresponsive) score, and the use of any supplemental oxygen.
- Outcome is assessed at midday on the day after the initial assessment and can be (ie, discharged, admitted to hospital, admitted to high care or intensive care, or deceased).

### Withdrawal of Individual Participants

If they wish, participants can leave the study at any time for any reason without any consequences. The investigator can withdraw a participant from the study if they consider it is in the participant’s best interests, such as avoiding delay or compromise of urgent medical care.

### Statistical Analysis

#### Sample Size Calculation

Due to the use of the Flash Mob Research design a formal sample size calculation is not possible. We assume that a number of 40 participating hospitals in Europe screening up to 50 patients each would result in a sample size of at least 2000 patients screened. This sample size should be able to answer questions about the proportion of the number of patients presenting with wearable devices and about changes in key vital signs between assessment in the hospital and a period 24 hours 1 week or 1 month previous.

#### Data Analysis

Data will be analyzed using SPSS (version 28; IBM Statistics). All variables will be assessed for normality. This study will use basic epidemiological data to determine if patient-owned monitoring devices can be used in an emergency care setting to assess the patient’s current severity of illness and compare it with their usual state of health.

Continuous data will be analyzed using the unpaired *t* test (if the data are normally distributed) or the Wilcoxon rank sum test (if the data are not normally distributed). Changes between measurements will be described as percentage change from the first measurement taken at home before admission as the assumed baseline and as an absolute change. Categorical data will be analyzed using chi-square test. Basic statistics and descriptives will be used to identify the distribution of patient and device characteristics. Missing data will not be imputed.

Associations between the baseline measurements and the specified clinical end points and the absolute and proportional change of physiological measurements and the specified clinical end points will be assessed using linear regression and logistic regression.

Patient characteristics will be expressed as absolute numbers per (sub)category for the total patient population, the discharged patients, and the admitted patients. The heart rate and step count data will be compared for their respective average or median for the different time points. Both will be tested for significant interaction between time, disposition, and change in either heart rate or step count using a generalized linear model.

For secondary exploratory analyses, the correlation between potential outcomes will be tested using Pearson coefficient correlation analysis (*r*), where the following interpretation will be used: below 0.10 negligible, between 0.10 and 0.39 weakly correlated, between 0.40 and 0.69 moderately correlated, between 0.70 and 0.89 strongly correlated, and above 0.89 very strongly correlated. The percentile change in heart rate and step count will be calculated in comparison to the baseline, which will be assumed to be at 30 days before a hospital visit.

#### Primary Outcome Measures

The primary outcome of the study will be feasibility as assessed by the proportion of eligible patients with emergency presentations who have devices as well as recruitment and retention rates.

#### Secondary Outcome Measures

Secondary outcomes measures include measures of epidemiology and physiology:

We will report trends for heart rate and step count, specifically the difference between the heart rate at home 24 hours, 1 week, and 1 month before clinical assessment from the wearable device compared with the first vital signs measured on admission to acute care as both an absolute and a proportional change.

We will also describe the degree of association between changes to vital signs from the community to assessment in acute care services with admission to a general ward or admission to a critical care area or discharge home within 24 hours from the time of the first hospital assessment.

The population wearing or carrying devices will be described in terms of gender, age, ethnicity, level of education, and digital literacy and contrasted with data from national surveys.

### Administrative Aspects, Monitoring, and Publication

#### Handling and Storage of Data and Documents

Data will be anonymously recorded in a research database (ie, Castor Electronic Data Capture System) in line with local data protection regulations, including but not limited to European General Data Protection Regulations (GDPR).

The collected data will be subsequently stored on a digital research environment from the Erasmus MC, a secure server with 2-factor authentication. There are no patient identifiers within the data. Only selected investigators of this study will have access to the server with the data. Data will be stored for 15 years.

#### Amendments

Amendments are changes made to the research after a favorable opinion by the accredited MEC has been given. All amendments will be notified to the ethics committee that gives a favorable opinion.

#### Annual Progress Report

The sponsor or investigator will submit a summary of the progress of the trial to the accredited MEC (Medisch Ethische Toetsings Commissie, METC) once the study is completed or, at the latest, after 1 year. Information will be provided on the date of inclusion of the first participant, the numbers of participants included and the numbers of participants that have completed the trial, serious adverse events or serious adverse reactions, other problems, and amendments.

#### Temporary Halt and (Premature) End of Study Report

The investigator or sponsor will notify the accredited METC of the end of the study within a period of 8 weeks. The end of the study is defined as the last patient’s last visit.

The sponsor will notify the METC immediately of a temporary halt of the study, including the reason of such an action.

In case the study is ended prematurely, the sponsor will notify the accredited METC within 15 days, including the reasons for the premature termination.

Within 1 year after the end of the study, the investigator or sponsor will submit a final study report with the results of the study, including any publications or abstracts of the study, to the accredited METC.

#### Public Disclosure and Publication Policy

The results of this study will be published in one or more peer-reviewed articles. Publications will be coordinated by the investigators or sponsors. Authorship of publications will be determined in accordance with the requirements published by the International Committee of Medical Journal Editors and in accordance with the requirements of the respective medical journal.

#### Benefits and Risks Assessment and Group Relatedness

There are no benefits or risks for participants of this study. The burden of participation in this study is low.

### Ethical Considerations

#### Regulation Statement

The study will be conducted in compliance with the principles of the Declaration of Helsinki (version 10, October 19, 2013) and the principal of Good Clinical Practice. Participants are not subject to procedures or required to follow rules of behavior and, as no additional data are collected the Medical Research Involving Human Subjects Act (also known by its Dutch abbreviation WMO) is not applicable to this study. Each principal investigator is responsible for ensuring that the study is performed in accordance with the protocol and Good Clinical Practice.

The review board of the Erasmus University Medical Center, Erasmus MC in Rotterdam, the Netherlands, has reviewed the research proposal. It confirmed that the rules laid down in the WMO, do not apply to this research proposal Medical Ethics Committee (MEC-2022-0795). The protocol (V1.2 17 February 2025) passed the required approvals in the United Kingdom Integrated Research Application System (IRAS 321129).

#### Recruitment and Consent

Patients eligible for inclusion are asked if they use a smartwatch or other wearables that measure heart rate by a collaborator of the study. If they do they fulfill the inclusion criteria, and they will then be informed about the study procedures and asked to participate after they have read the patient information form. After receiving this form patients will be asked if they will agree to sign written informed consent.

#### Governance

Hospitals will be recruited across Europe and coordinated by the study steering board. The study steering board consists of the principal investigators and members of the Safer home consortium. Centers are encouraged to collect data on a small cohort of patients before the main study week (May 10-22, 2024). During the main study week, all participating units are asked to collect data on all patients presenting in the study areas during a 24-hour period.

Participating countries will be responsible for their own submission for approval and coordination of research.

## Results

Approvals have been completed in 11 sites in Denmark, 17 sites in the Netherlands, 1 site in Switzerland, and 7 sites in the United Kingdom.

The main FUSE study has gone live on the May 15, 2024, in 34 hospitals in the Netherlands (n=17), the United Kingdom (n=7), Denmark (n=9), and Switzerland (n=1). A total of 1137 patients were screened, of these, 452 patients were included and 209 patients had data on at least 2 collection points, allowing for an analysis of trends.

Preliminary analysis will be completed by December 31, 2024, and the main manuscript will be submitted for publication by the end of March 2025.

## Discussion

### Anticipated Findings

While sensors that can be carried or worn are in widespread use with a project market size in 2023 of in excess of US $4 billion [20], previous research has found little evidence of their use in emergency care [21]. This study aims to determine if there are opportunities to use the data generated by these consumer-held devices to improve patient care. Trends in vital signs and mobility are used to quantify risks to patients and are likely to become more important as the proportion of patients with chronic conditions (and abnormal vital signs during signs of clinical stability) is increasing. For clinical use we anticipate a number of use cases: Assessing patients whose vital signs have not changed in the week previous presentation in an acute care setting might reassure clinicians that the condition is overall stable and that treatment in the community is likely to be safe. Assessing patients whose vital signs have significantly changed in the days or hours before presentation might increase the confidence of clinicians to admit for observation and treatment in the hospital. This type of decision-making is commonly undertaken during an initial observation phase in emergency departments or acute medical unit with patients being observed for a night before committing to a decision about admission or discharge. We anticipate that this type of decision-making could be undertaken during the initial presentation if data on markers of physiological instability is available already at this point.

### Limitations

The distribution of wearables in the population might point toward the exclusion of a significant proportion of the population from this type of support: patients who are less financially well off or not sufficiently digitally or health literate might be disadvantaged in a future health care. This data will hence be collected as part of FUSE. While this study focuses on readily available data and will require in future supplementation by detailed qualitative data.

The study is conducted by a group of secondary care clinicians with expertise in medical emergencies. Many of the patients who will be screened and included will have previously presented in primary care. There is no theoretical reason that the results from this study might not be applicable to those presenting in primary care, but this might require future research.

In preparation of the study, we reviewed sales for the top 20 devices in the Dutch and British markets. For these devices, we have created a data dictionary for investigators that will enable them to assist patients in the location of relevant data. With new devices coming online and changes in consumer habits, the frequency distribution of these devices might however be soon outdated.

The study will explicitly not address the quality of data collected in wearables. We will, however, report on the distribution of devices and approval for medical use where applicable. In the same vein, we anticipate that the duration of usage by individuals will affect the measurements. This is something that we might not be able to evaluate in the current setup.

Given that patients will use a range of devices with different methods for data acquisition and different usage patterns our analysis will focus on the comparison of relative changes in parameters for individuals (intraindividual variation). This relies on the assumption that individuals will have a pattern of usage that is constant over time. Further research will be required, especially if it turns out that the variation between the collected parameters turns out to be large in the days before deterioration and presentation to the hospital.

Finally, future health care needs to be equitable. FUSE will include data that will allow us to gauge some aspects of equity, diversity, and inclusion which are crucial for the development of policy and strategy in this field.

### Study Impact

Given the funding shortfall in most health economies consumer-owned monitoring will represent a tempting addition for health care providers in primary and secondary care. The supporting tools such as the wearable device dictionary (Multimedia Appendix 2) might be useful to clinicians even without the data from the study.

### Conclusions

This article describes a feasibility study. The results should aid power calculations and the design of more definitive observational and ultimately interventional trials.

From a health policy point of view, there is concern that, at present, device ownership might be more common in those who are younger and well off, thus potentially amplifying problems around equitable access to health care and emphasizing the inverse care law. The FUSE study and subsequent research should provide data that might direct manufacturers toward the design of devices that are suitable for the widest range of users. We hope that FUSE will help policymakers and clinical teams in the design of better and safer services around digitally enabled patients and for those who are not (yet) connected to mHealth systems.

## Appendix

Multimedia Appendix 1 Sample consent form.

Multimedia Appendix 2 Wearable devices data guide for investigators of the FUSE study.

Multimedia Appendix 3 Spirit checklist for study protocols.

## Abbreviations

DECAF: Dyspnea, Eosinopenia Consolidation, Acidemia, and Atrial Fibrillation
GDPR: General Data Protection Regulations
IRAS: Integrated Research Application System
MEC: Medical Ethics Committee
WMO: Medical Research Involving Human Subjects Act

## References

[1] van der Kluit MJ, Dijkstra G, de Rooij SE (2018). The decision-making process for unplanned admission to hospital unveiled in hospitalised older adults: a qualitative study. BMC Geriatr.

[2] Emmanuel A, Ismail A, Kellett J (2010). Assessing the need for hospital admission by the cape triage discriminator presentations and the simple clinical score. Emerg Med J.

[3] Subbe CP, Jishi F, Hibbs RAB (2010). The simple clinical score: a tool for benchmarking of emergency admissions in acute internal medicine. Clin Med (Lond).

[4] Kellett J, Emmanuel A, Deane B (2011). Who will be sicker in the morning? Changes in the simple clinical score the day after admission and the subsequent outcomes of acutely ill unselected medical patients. Eur J Intern Med.

[5] Hosseini M, Baikpour M, Yousefifard M, Fayaz M, Koohpayehzadeh J, Ghelichkhani P, Asady H, Asgari F, Etemad K, Rafei A, Gouya MM (2015). Blood pressure percentiles by age and body mass index for adults. EXCLI J.

[6] Chester JG, Rudolph JL (2011). Vital signs in older patients: age-related changes. J Am Med Dir Assoc.

[7] Berge HM, Isern CB, Berge E (2015). Blood pressure and hypertension in athletes: a systematic review. Br J Sports Med.

[8] Bleyer AJ, Vidya S, Russell GB, Jones CM, Sujata L, Daeihagh P, Hire D (2011). Longitudinal analysis of one million vital signs in patients in an academic medical center. Resuscitation.

[9] Jones M (2012). NEWSDIG: the national early warning score development and implementation group. Clin Med (Lond).

[10] Lim WS, van der Eerden MM, Laing R, Boersma WG, Karalus N, Town GI, Lewis SA, Macfarlane JT (2003). Defining community acquired pneumonia severity on presentation to hospital: an international derivation and validation study. Thorax.

[11] Blatchford O, Murray WR, Blatchford M (2000). A risk score to predict need for treatment for upper-gastrointestinal haemorrhage. Lancet.

[12] Echevarria C, Steer J, Heslop-Marshall K, Stenton SC, Hickey PM, Hughes R, Wijesinghe M, Harrison RN, Steen N, Simpson AJ, Gibson GJ, Bourke SC (2016). Validation of the DECAF score to predict hospital mortality in acute exacerbations of COPD. Thorax.

[13] Grant S, Crimmons K (2018). Limitations of track and trigger systems and the national early warning score. Part 2: sensitivity versus specificity. Br J Nurs.

[14] Brekke IJ, Puntervoll LH, Pedersen PB, Kellett J, Brabrand M (2019). The value of vital sign trends in predicting and monitoring clinical deterioration: a systematic review. PLoS One.

[15] Brabrand M, Kellett J, Opio M, Cooksley T, Nickel CH (2018). Should impaired mobility on presentation be a vital sign?. Acta Anaesthesiol Scand.

[16] Smartphone ownership in the US by age 2015-2023. Statista.

[17] Smartphone ownership by UK age 2012-2024. Statista.

[18] Alsma J, van Saase JLCM, Nanayakkara PWB, Schouten WEMI, Baten A, Bauer MP, Holleman F, Ligtenberg JJM, Stassen PM, Kaasjager KHAH, Haak HR, Bosch FH, Schuit SC, FAMOUS Study Group* (2017). The power of flash mob research: conducting a nationwide observational clinical study on capillary refill time in a single day. Chest.

[19] Rockwood K, Song X, MacKnight C, Bergman H, Hogan D, McDowell I, Mitnitski A (2005). A global clinical measure of fitness and frailty in elderly people. CMAJ.

[20] (2022). Wearable sensors market size to surpass USD 7.5 Bn By 2030. PrecedenceResearch.

[21] Hamza M, Alsma J, Kellett J, Brabrand M, Christensen EF, Cooksley T, Haak HR, Nanayakkara PW, Merten H, Schouten B, Weichert I, Subbe CP (2021). Can vital signs recorded in patients' homes aid decision making in emergency care? A scoping review. Resusc Plus.

