# Supplementary material for: Feasibility of Using Resting Heart Rate and Step Counts From Patient-Held Sensors During Clinical Assessment of Medical Emergencies (FUSE): Protocol for Prospective Observational Study in European Hospitals
Source: JMIR Res Protoc. 2025 Apr 28;14:e55975. doi: 10.2196/55975 (PMC12070009; doi:10.2196/55975)
Supplement: Multimedia Appendix 1 [file resprot_v14i1e55975_app1.pdf]

Participant Information Leaflet

Feasibility of Using resting heart rate and step-counts from patient-held Sensors during clinical assessment of medical Emergencies (FUSE-Study)

Many thanks for your interest in this research project, investigating the use of wearable technology to assess changes in vital signs.

Increasing numbers of people are using wearable technology, such as smartwatches and fitness trackers, to monitor vital signs, including heart rate and activity levels. Changes in your vital signs are compared to ‘normal’ ranges from a healthy resting population, however, it may be more appropriate to compare this with your own normal values when you are well. Knowing the values of your vital signs whilst well might help clinicians to better understand changes from your baseline and help in assessing the severity of illness.

Why am I being asked to take part?

We are interested in assessing changes in data on vital signs, such as heart rate and activity levels, that are recorded on a wearable monitoring device, such as smartwatch or fitness tracker. We want to compare the levels that are recorded by your medical team today with your own baseline levels from 1 day and 1 week prior to this, and then assess the outcome of your presentation to hospital.

What does the study involve?

You will be asked to locate your vital signs data on your smartwatch or smartphone and inform the clinical investigator of the values prior to your presentation to the hospital. These will be recorded by the clinical investigator, and other information including your gender, ethnicity, date of birth, location, literacy, and the type of wearable device that you use will also be recorded.

7-days after this, the investigator will access your hospital record to document the outcome of your presentation to hospital, such as admission or discharge home.

What are the benefits to taking part?

You will be contributing to important research which could help develop a better way to identify patients at risk of severe illness. You will also be helping us to better understand how wearable technology could be used in healthcare to improve patient care.

What are the risks of taking part?

We anticipate that taking part in this study is generally very safe, and there are no identified risks of taking part. There will be no delay in emergency treatment as a result of taking part.

What happens after I take part?

We will not need to contact you following your involvement in this study.

The research team will need to access your hospital record to document whether you stayed in hospital or were discharged home in the 7 days after the study.

All patient data used will be anonymised, and we will not access your medical record for reasons other than those outlined.

Expenses and payments

There are no payments for taking part in this study.

What if there is a problem?

If you have a concern about any aspect of this study, you should ask to speak to the researchers or the hospital concerns team who will do their best to answer your questions (Concerns office, telephone 01248-384 194).

In case of accidental disclosure of confidential details, you will be informed and the disclosure will be reported in line with Standard Operating Procedures of BCUHB.

Will my taking part in the study be kept confidential?

For the study we will ask you a few questions. One copy of the answers to those questions will be added to your hospital notes and might help your doctors and nurses to look after you. All information which is collected about you during the course of the research will be kept strictly confidential. Any quotes from you will be anonymized.

Any information about you that is not in the hospital notes will have your name, address and any other personal details removed so that you cannot be recognized. Your confidentiality will be safeguarded during and after the study by the Caldicott principles and/or Data Protection Act 1998. All identifiable information will be deleted 12 months after the completion of the study.

What happens if I lose my ability to consent whilst the study is ongoing?

If for any reason you lose the capacity to consent after signing the consent form and taking part in the study today, your records will still be accessed at 7 days on the basis of the consent already provided.

How will my information be used as part of the study?

We will need to use the following information from you, and from your medical records for this research project:

- Your name
- Date of Birth
- Ethnicity
- Gender
- The degree or level of school you have completed
- Your level of confidence of using a digital device

- The type of your wearable device
- Heart rate and walking distance 1 week and 1 day prior to current admission and at the day of your current admission
- Current heart rate and NEWS score from your clinical records
- The outcome of your care, including admission to hospital or discharge home in the 7 days after the test

People will use this information to do the research or to check your records to make sure that the research is being done properly.

People who do not need to know who you are will not be able to see your name or contact details. Your data will have a code number instead.

We will keep all information about you safe and secure.

Some of your anonymous information will be shared with other NHS trusts in the UK. They must follow our rules about keeping your information safe.

Once we have finished the study, we will keep some of the data so we can check the results. We will write our reports in a way that no-one can work out that you took part in the study.

What will happen if I don't carry on with the study?

You can stop being part of the study at any time, without giving a reason.

We need to manage your records in specific ways for the research to be reliable. This means that we won't be able to let you see or change the data we hold about you.

If you agree to take part in this study, you will have the option to take part in future research using your anonymised data saved from this study. For more information about how your information will be used as part of this study you can contact our Data Protection Officer at bcu.dpo@wales.nhs.uk, contact our Information Governance Department on 01978 727689, or speak to one of the research team.

Further information can also be found at www.hra.nhs.uk/information-about-patients/ and www.hra.nhs.uk/patientdataandresearch.

What will happen to the results of the research study?

The results of the study will be published in medical journals. You will not be identified in any report/publication.

Who is organizing or sponsoring the research?

The study is sponsored by BCUHB. The researchers have not received extra funding for undertaking the study. Funding for the administration of the study has been received by the Society for Acute Medicine.

Further information and contact details:

We hope that you are interested in working with us on this important project.

For more information please don't hesitate to e-mail us on Christian.Subbe@Wales.NHS.UK.

Consent

We ask you for your permission to participate in test and the review of your medical information.

You are free to stop participating at any time and without giving any reason to withdraw your permission. This has no negative consequences for your treatment.

If, after reading these notes questions remain unanswered, please send them directly to Christian.Subbe@wales.nhs.uk.
