# Supplementary material for: Feasibility of Using Resting Heart Rate and Step Counts From Patient-Held Sensors During Clinical Assessment of Medical Emergencies (FUSE): Protocol for Prospective Observational Study in European Hospitals
Source: JMIR Res Protoc. 2025 Apr 28;14:e55975. doi: 10.2196/55975 (PMC12070009; doi:10.2196/55975)
Supplement: Multimedia Appendix 2 [file resprot_v14i1e55975_app2.pdf]

Apple

Data available on **Apple Health** app

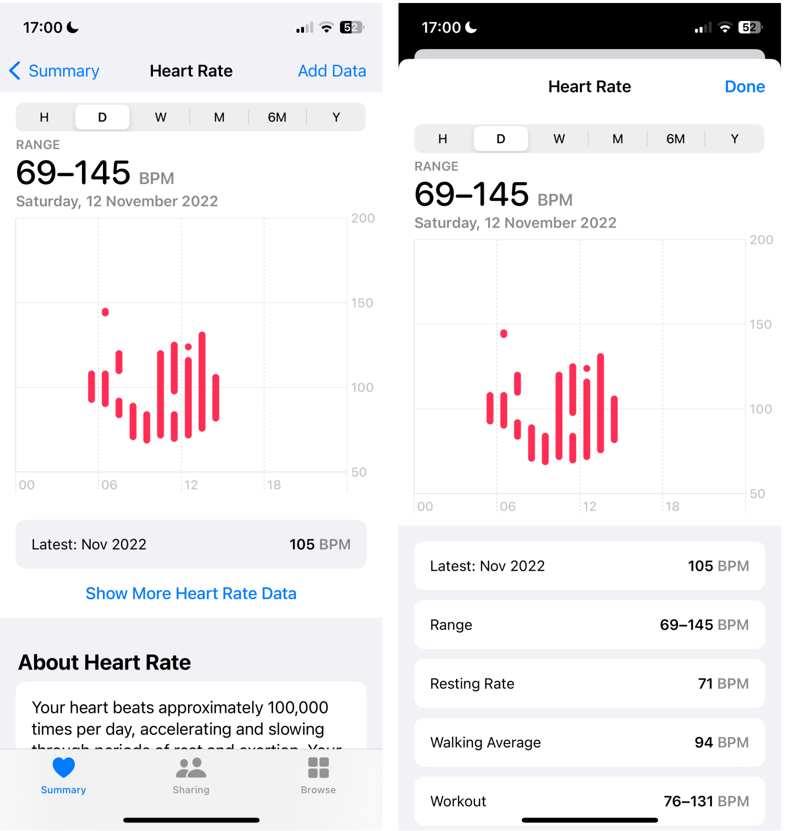

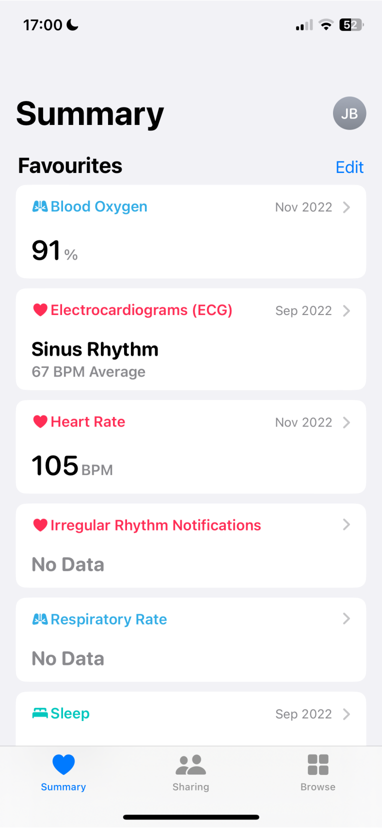

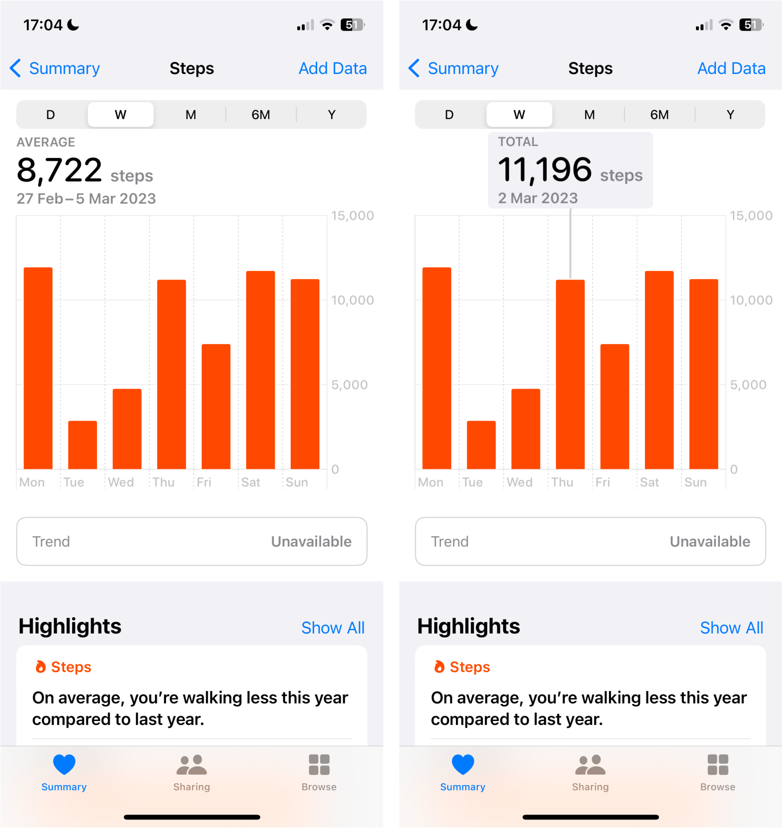

Heart rate data: range, average, resting average, walking average

Step count data: daily/hourly total steps, weekly/monthly/6-monthly/yearly daily average

Apple

Data available on **Apple Health** app

	Steps	HR	ECG	Irregular HR Warning	O_2 Sats	Temp	Perspiration	Sleep Tracker	Fall Detection
**Watch Ultra**	✓	✓	✓	✓	✓	✓	X	✓	✓
**Watch Series 8**	✓	✓	✓	✓	✓	✓	X	✓	✓
**Watch Series 7**	✓	✓	✓	✓	✓	X	X	✓	✓
**Watch Series 6**	✓	✓	✓	✓	✓	X	X	✓	✓
**Watch Series 3**	✓	✓	X	✓	X	X	X	✓	X
**Watch SE (2022)**	✓	✓	X	✓	X	✓	X	✓	✓

Fitbit

Data available on **Fitbit** app

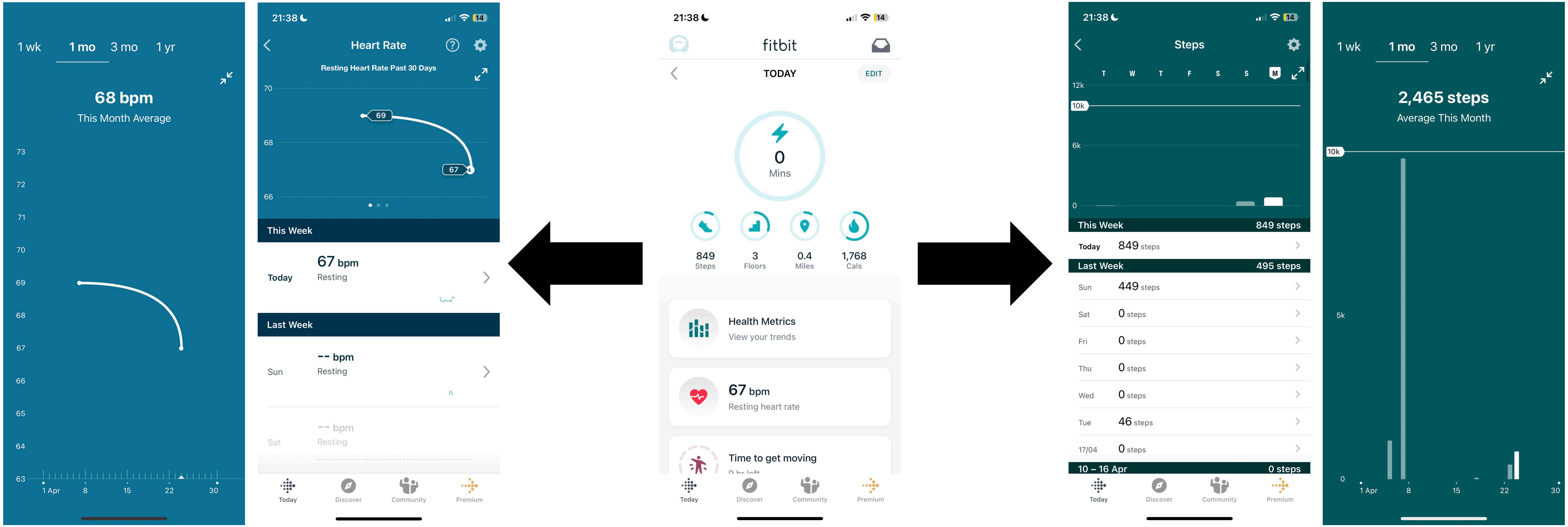

Heart rate data: resting average, weekly/monthly/3-monthly/yearly daily resting average

Step count data: daily/weekly total steps, weekly/monthly/3-monthly/yearly daily average

Fitbit

Data available on **Fitbit** app

	Steps	HR	ECG	Irregular HR Warning	O_2 Sats	Temp	Perspiration	Sleep Tracker	Fall Detection
**Sense 2**	✓	✓	✓	✓	✓	✓	✓	✓	X
**Sense**	✓	✓	✓	✓	✓	✓	X	✓	X
**Versa 4**	✓	✓	X	X	✓	X	X	✓	X
**Versa 3**	✓	✓	X	X	✓	✓	X	✓	✓
**Versa 2**	✓	✓	X	X	✓	X	X	✓	✓
**Charge 5**	✓	✓	✓	✓	✓	✓	✓	✓	X
**Luxe**	✓	✓	X	X	✓	X	X	✓	X
**Inspire 3**	✓	✓	X	✓	✓	✓	X	✓	X
**Inspire 2**	✓	✓	X	X	X	X	X	✓	X

Garmin

Data available on **Garmin Connect** app

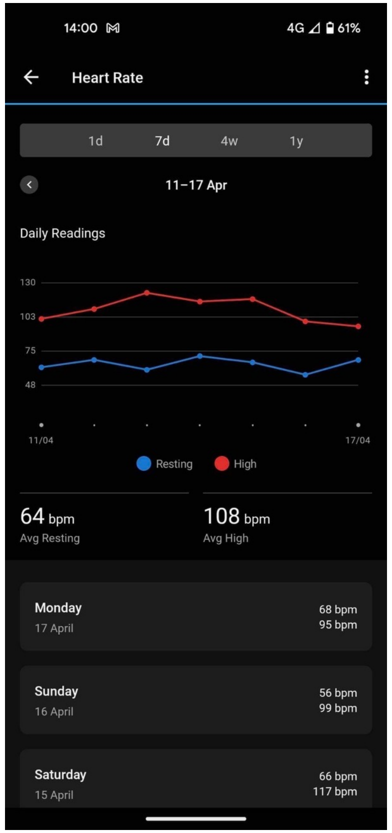

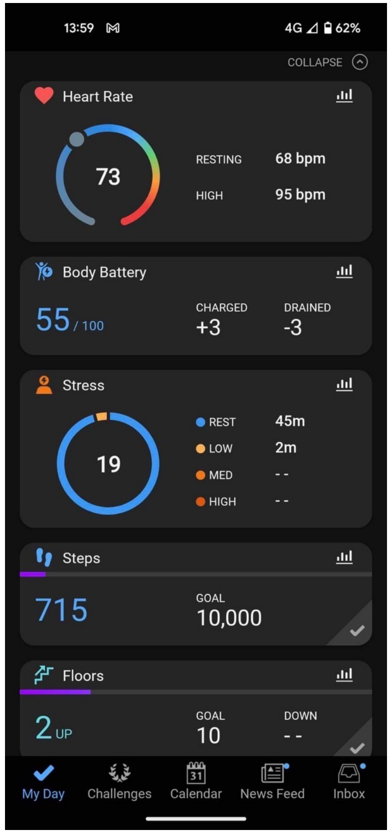

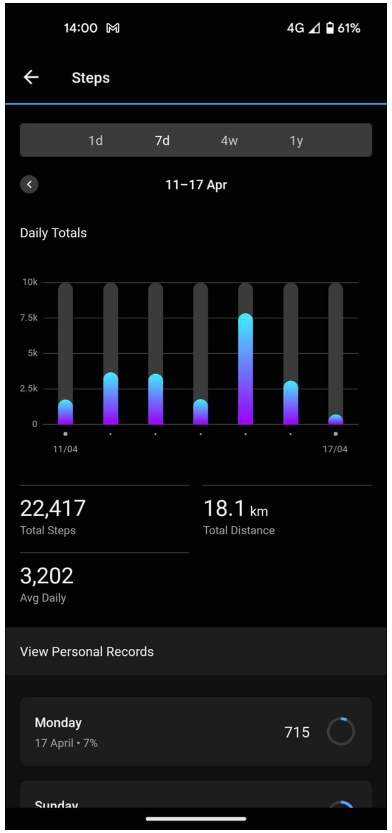

Heart rate data: range, resting average, walking average

Step count data: daily/hourly total steps, daily/weekly/monthly/yearly daily average

Garmin

Data available on **Garmin Connect** app

	Steps	HR	ECG	Irregular HR Warning	O_2 Sats	Temp	Perspiration	Sleep Tracker	Fall Detection
**Venu Sq 2**	✓	✓	X	✓	✓	X	X	✓	X
**Venu Sq**	✓	✓	X	X	✓	X	X	✓	X
**Venu 2**	✓	✓	X	✓	✓	✓	X	✓	X
**Venu 2 Plus**	✓	✓	✓	✓	✓	✓	X	✓	✓
**Forerunner 945**	✓	✓	X	✓	✓	✓	X	✓	✓
**Forerunner 265**	✓	✓	X	✓	✓	✓	X	✓	✓
**Forerunner 745**	✓	✓	X	✓	✓	✓	X	✓	X
**Forerunner 245**	✓	✓	X	✓	✓	X	X	✓	✓
**Forerunner 55**	✓	✓	X	✓	✓	X	X	✓	✓

Garmin

Data available on **Garmin Connect** app

	Steps	HR	ECG	Irregular HR Warning	O_2 Sats	Temp	Perspiration	Sleep Tracker	Fall Detection
**Forerunner 45**	✓	✓	X	X	X	X	X	✓	✓
**Instinct Crossover**	✓	✓	X	✓	✓	✓	✓	✓	✓
**Instinct 2**	✓	✓	X	✓	✓	✓	✓	✓	✓
**Instinct**	✓	✓	X	✓	X	✓	X	✓	X
**Fenix 7**	✓	✓	X	X	✓	✓	X	✓	✓
**Fenix 6 Pro**	✓	✓	X	✓	✓	✓	X	✓	✓
**Fenix 6**	✓	✓	X	✓	✓	✓	X	✓	X
**Vivoactive 4**	✓	✓	X	✓	✓	✓	X	✓	X
**Vivosmart 5**	✓	✓	X	X	✓	X	X	✓	X

Samsung

Data available on **Samsung Health** app

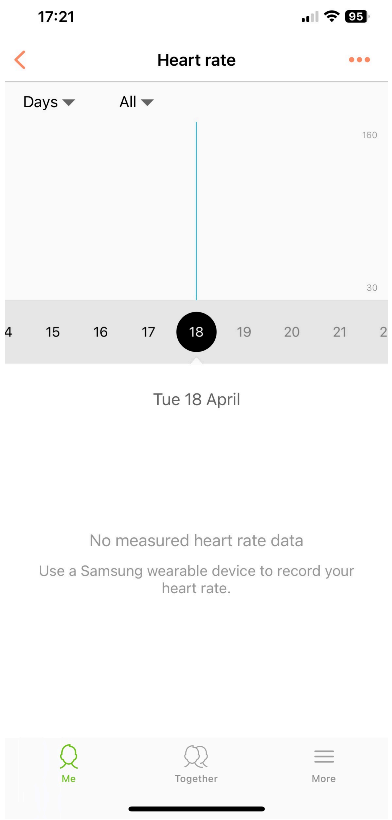

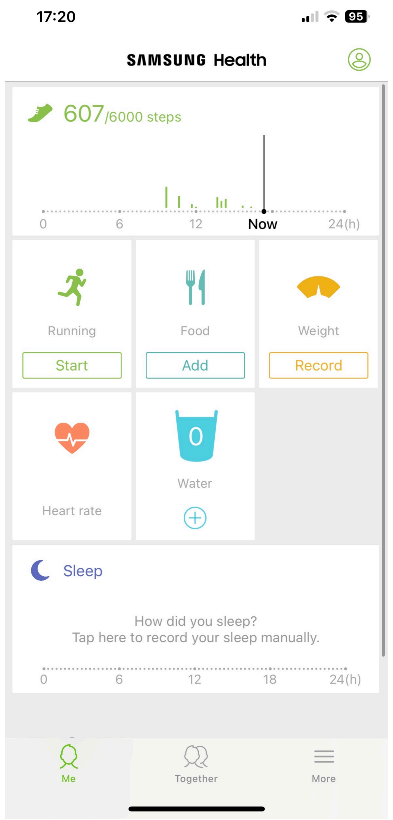

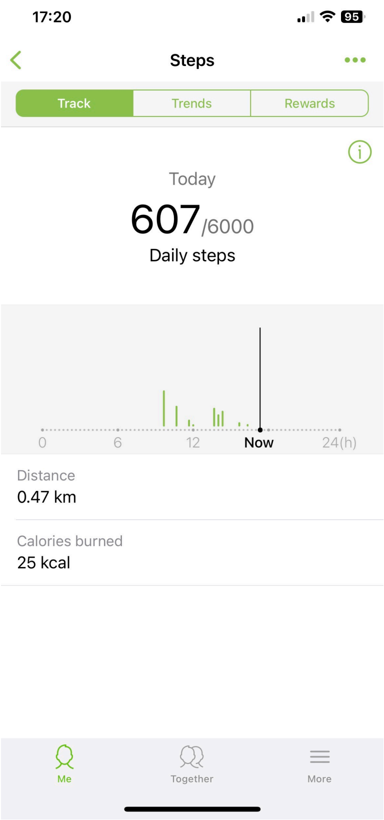

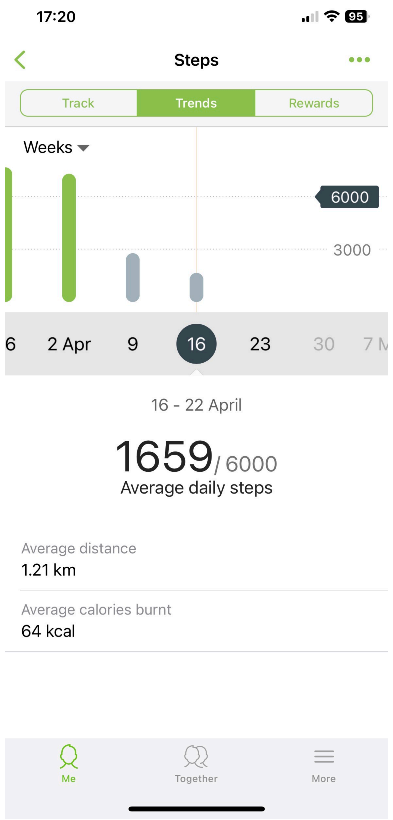

Heart rate data

Step count data: daily/hourly total steps, daily/weekly/monthly daily average

Samsung

Data available on **Samsung Health** app

	Steps	HR	ECG	Irregular HR Warning	O_2 Sats	Temp	Perspiration	Sleep Tracker	Fall Detection
Galaxy Watch5 Pro	✓	✓	✓	✓	✓	✓	X	✓	✓
Galaxy Watch5	✓	✓	✓	✓	✓	X	X	✓	✓
Galaxy Watch4 Classic	✓	✓	✓	✓	✓	X	X	✓	✓
Galaxy Watch4	✓	✓	✓	✓	✓	X	X	✓	✓

Huawei

Data available on **Huawei Health** app

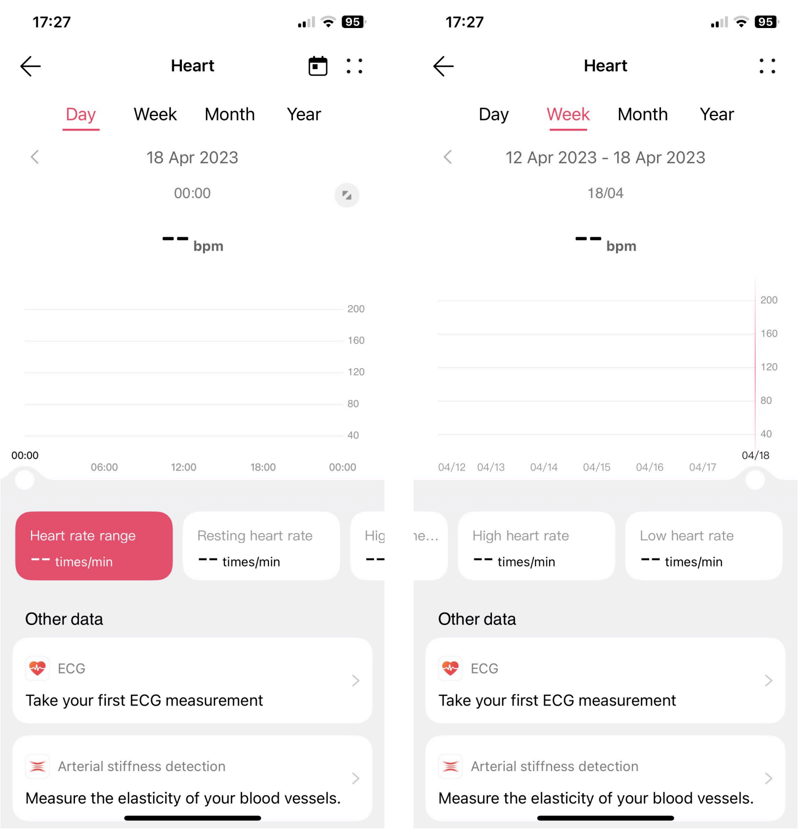

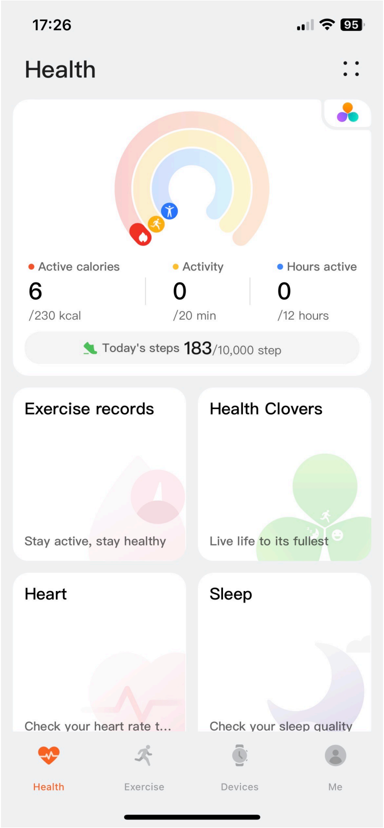

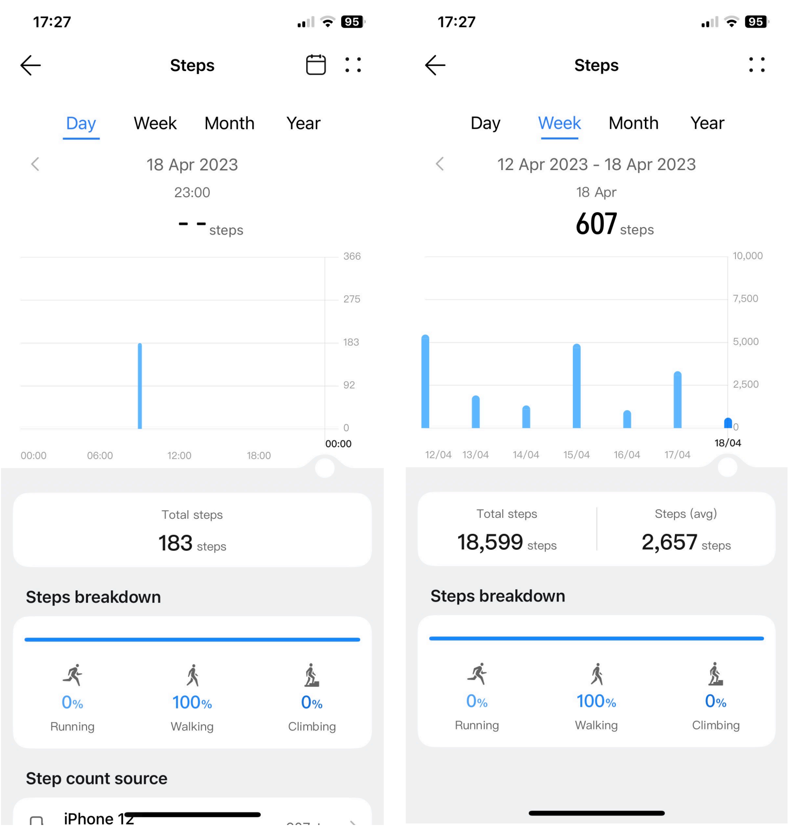

Heart rate data: range, average, resting average, daily/weekly/monthly average

Step count data: daily/hourly total steps, daily/weekly/monthly/yearly daily average

Huawei

Data available on **Huawei Health** app

	Steps	HR	ECG	Irregular HR Warning	O_2 Sats	Temp	Perspiration	Sleep Tracker	Fall Detection
Band 7	✓	✓	X	X	✓	X	X	✓	X
Band 6	✓	✓	X	✓	✓	X	X	✓	X
Watch GT3 Pro	✓	✓	✓	✓	✓	✓	X	✓	X
Watch GT3 SE	✓	✓	X	X	✓	X	X	✓	X
Watch GT3	✓	✓	X	✓	✓	✓	X	✓	X
Watch GT Runner	✓	✓	X	X	✓	X	X	✓	X
Watch GT2	✓	✓	X	✓	✓	X	X	✓	X
Watch GT2 Pro	✓	✓	X	✓	✓	X	X	✓	X
Watch GT	✓	✓	X	X	X	X	X	✓	X
Watch Fit 2	✓	✓	X	✓	✓	✓	X	✓	X
